# Validation of perfusion dyssynchrony indices as a novel non-invasive diagnostic tool in the detection of hemodynamically significant coronary artery disease in the setting of three dimensional (3D) myocardial perfusion CMR

**DOI:** 10.1186/1532-429X-18-S1-P86

**Published:** 2016-01-27

**Authors:** Joy S Shome, Roy Jogiya, Adriana Villa, Eva Sammut, Divaka Perera, Simon Redwood, Sebastian Kozerke, Sven Plein, Amedeo Chiribiri

**Affiliations:** 1 Cardiovascular Imaging, St. Thomas' Hospital, Rayne Institute, King's College London, London, UK; 2 Cardiovascular Division, St. Thomas' Hospital, Rayne Institute, King's College London, London, UK; 3 Institute for Biomedical Engineering University and ETH Zurich, Zurich, Switzerland; 4 Multidisciplinary Cardiovascular Research Centre & Division of Biomedical Imaging, Leeds Institute of Cardiovascular and Metabolic Medicine, University of Leeds, Leeds, UK

## Background

Perfusion dyssynchrony analysis provides a novel insight into the evaluation of myocardial ischaemia due to coronary artery disease (CAD). Perfusion dyssynchrony indices measure differences in the temporal distribution of the wash-in of contrast agents across the left ventricular wall. In a previous 2D study the temporal dyssynchrony of LV perfusion was measured using four indices - variance and coefficient of variation of the time to maximum signal upslope (V-TTMU and C-TTMU), and variance, and coefficient of variation of the time to peak myocardial signal enhancement (V-TTP and C-TTP).{Chiribiri:2014en} TTP detected CAD better than TTMU, with C-TTP being the most sensitive.

3D myocardial perfusion CMR provides the advantage of whole heart coverage at the expense of inferior in plane spatial resolution. Our aim was to validate perfusion dyssynchrony analysis on 3D perfusion datasets to detect significant CAD.

## Methods

25 patients were retrospectively identified.{Jogiya:2012dq} All patients underwent rest and adenosine-stress first pass 3D whole heart myocardial perfusion CMR at 3T, and invasive angiography with fractional flow reserve (FFR) of lesions of visual stenosis severity >50% (FFR <0.75 considered as hemodynamically significant). Patients were classified according to the number of vessels with hemodynamically significant CAD.

Perfusion dyssynchrony analysis was performed on 3D stress images using indices C-TTP and V-TTP, which on 2D datasets were the most accurate and reproducible perfusion dyssynchrony indices.

## Results

4 patients did not have significant CAD, 11 had single vessel disease (SVD), and 10 had multivessel disease (MVD).

Mean C-TTP and V-TTP values for groups without significant CAD, with SVD, with MVD, and with either SVD or MVD were 7.7 ± 1.3, 0.66 ± 0.08; 13.5 ± 5.4, 4.2 ± 4.6; 14.8 ± 5.0, 5.3 ± 3.6; and 14.1 ± 5.2, 4.7 ± 4.1 respectively.

T-test was used for statistical analysis. Compared to patients without significant CAD, both C-TTP and V-TTP values were significantly higher in SVD group (p < 0.01), MVD group (p = 0.001), and with both SVD and MVD groups combined (p < 0.0001). Comparing SVD with MVD, values for both indices were marginally different and not statistically significant.

## Conclusions

Our study validates the concept of perfusion dyssynchrony and demonstrates its feasibility on 3D perfusion datasets. C-TTP and V-TTP showed consistency in detecting functionally significant CAD. Patients without significant CAD had relatively low dyssynchrony, with a steep and consistently significant increase in dyssynchrony in significant CAD patients. Values were slightly higher in MVD compared to SVD but not statistically significant.

We anticipate that with a larger sample size perfusion dyssynchrony indices may also help localise the presence of ischaemia in specific coronary territories.

**Figure 1:**
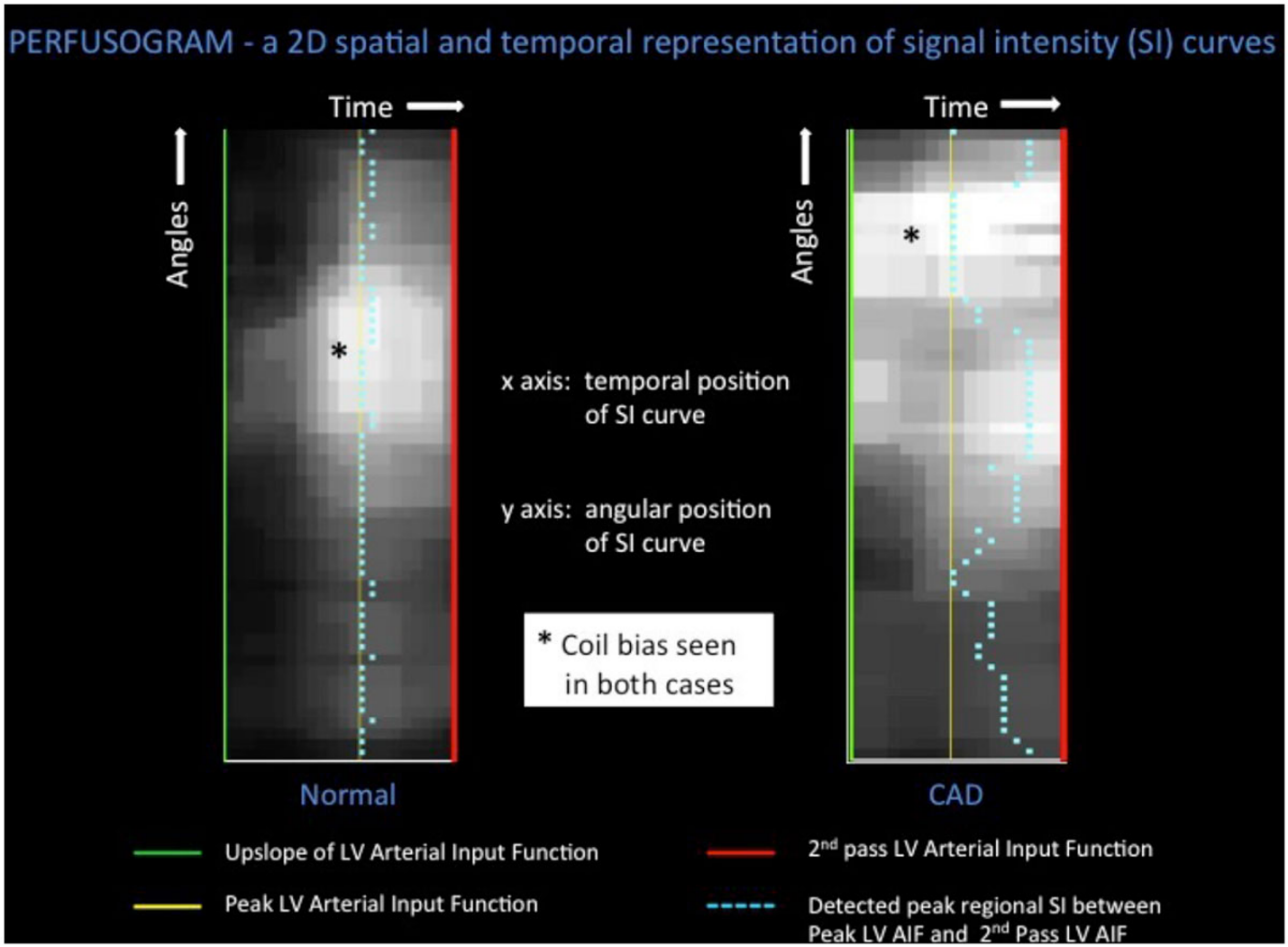
Figure 1

